# Commentary: Causal associations of gut microbiota and metabolites on sepsis: a two-sample Mendelian randomization study

**DOI:** 10.3389/fimmu.2024.1339176

**Published:** 2024-01-17

**Authors:** Jiamin Xu, Hongyan Zhang

**Affiliations:** Medical Center of Burn Plastic and Wound Repair, The First Affiliated Hospital, Jiangxi Medical College, Nanchang University, Nanchang, China

**Keywords:** sepsis, Mendelian randomization, causal associations, gut metabolites, gut microbiota

We read the paper “Causal associations of gut microbiota and metabolites on sepsis: a two-sample Mendelian randomization study” with great care published by Zhao et al. in Frontiers in Immunology (1). Sepsis is a globally significant cause of morbidity and mortality. Despite substantial progress in sepsis treatment, there remains a lack of definitive evidence supporting therapeutic interventions targeting underlying pathophysiological mechanisms to improve outcomes. In this study, the authors concluded that there are intricate interactions between the gut microbiota, particularly the genus Clostridium, and the metabolite α-hydroxybutyrate in the septic environment. Undoubtedly, the findings of this study are crucial. However, we have some concerns about the methods employed by Zhao et al. in their research.

Firstly, in enhancing the logical coherence and professional depth of research design, it is crucial to analyze the multidimensional characteristics of patient outcome data thoroughly. Notably, in assessing the diagnostic accuracy of sepsis as organ dysfunction caused by infection, adherence to the international consensus definition of sepsis-3, as published by JAMA, is essential in this context (2). The UK Biobank database utilizes the ICD-10 coding system, categorizing sepsis and septic shock as A41.9 and R57.2, respectively. However, solely relying on such codes for diagnosing sepsis has limitations, as they may not fully comply with the latest definition standards of Sepsis-3. Therefore, in reviewing sepsis outcome data, it is imperative to ensure that the data extraction process is aligned with the Sepsis-3 definition while accurately encompassing diagnostic information of septic shock to enhance the completeness and scientific validity of the research. This review process is vital in ensuring data quality, as reliance solely on ICD-10 codes may not comprehensively reflect the latest clinical practice standards, potentially leading to interpretive biases in research findings.

In this study, the researchers concluded based on two sepsis-related GWAS datasets, using total sepsis and sepsis 28-day mortality as outcomes. However, this may lead to misunderstanding. The temporal gap between the occurrence of gut microbiota and metabolites and the onset of sepsis needs careful consideration. Whether data were collected before the onset of sepsis to assess the relationship between the two is pivotal, as temporal disparities may impact the interpretation of study conclusions.

The study reveals exposures involving various gut microbiota and metabolites, which may play intermediate roles in sepsis development. Therefore, we believe that incorporating multivariable causal inference methods, such as multivariable Mendelian randomization, can mitigate the impact of intermediate effects on the independence of exposure and outcome (3).

Lastly, it appears that the authors did not account for confounding factors in this study. In Mendelian randomization analysis, ensuring that genetic polymorphisms are not confounding factors between the exposure and sepsis outcomes is crucial and constitutes one of the three core assumptions of MR (4). Therefore, we suggest that including a step to eliminate confounding factors would likely yield more reliable conclusions.

In summary, through Mendelian randomization research, the authors discovered close interactions between the gut microbiota, especially the genus Clostridium, and the metabolite α-hydroxybutyrate in the context of sepsis. These findings are commendable. However, further improvements in the research methods would contribute to a more robust study.

## Author contributions

JX: Conceptualization, Writing – original draft, Writing – review & editing. HZ: Funding acquisition, Supervision, Writing – original draft, Writing – review & editing.
